# Recent applications of retention modelling in liquid chromatography

**DOI:** 10.1002/jssc.202000905

**Published:** 2020-11-03

**Authors:** Mimi J. den Uijl, Peter J. Schoenmakers, Bob W.J. Pirok, Maarten R. van Bommel

**Affiliations:** ^1^ Analytical Chemistry Group van ’t Hoff Institute for Molecular Sciences, University of Amsterdam Amsterdam The Netherlands; ^2^ Centre for Analytical Sciences Amsterdam (CASA) Amsterdam The Netherlands; ^3^ University of Amsterdam Faculty of Humanities, Conservation and Restoration of Cultural Heritage Amsterdam The Netherlands

**Keywords:** lipophilicity determination, method optimization, method transfer, stationary‐phase characterization, retention mechanisms

## Abstract

Recent applications of retention modelling in liquid chromatography (2015–2020) are comprehensively reviewed. The fundamentals of the field, which date back much longer, are summarized. Retention modeling is used in retention‐mechanism studies, for determining physical parameters, such as lipophilicity, and for various more‐practical purposes, including method development and optimization, method transfer, and stationary‐phase characterization and comparison. The review focusses on the effects of mobile‐phase composition on retention, but other variables and novel models to describe their effects are also considered. The five most‐common models are addressed in detail, i.e. the log‐linear (linear‐solvent‐strength) model, the quadratic model, the log–log (adsorption) model, the mixed‐mode model, and the Neue–Kuss model. Isocratic and gradient‐elution methods are considered for determining model parameters and the evaluation and validation of fitted models is discussed. Strategies in which retention models are applied for developing and optimizing one‐ and two‐dimensional liquid chromatographic separations are discussed. The review culminates in some overall conclusions and several concrete recommendations.

**Article Related Abbreviations**
ADS: adsorption
AIC: Akaike information criterion
ANN: artificial neural network
GA: genetic algorithm
HILIC: hydrophilic‐interaction chromatography
HSM: hydrophobic‐subtraction model
IEX: ion‐exchange chromatography
LFER: linear‐free‐energy relationships
LSS: linear solvent strength
MM: mixed mode
NK: Neue–Kuss
NPLC: normal‐phase liquid chromatography
PAH: polycyclic aromatic hydrocarbon
Q: quadratic

## 1 INTRODUCTION

LC is one of the most essential and pervasive techniques in the toolbox of analytical chemists. Retention modeling serves as a useful technique available for analytical chemists to rapidly develop methods. In LC, an analyte is distributed between a non‐moving stationary phase and a moving mobile phase. The time it takes for the analyte to migrate through the column is referred to as the retention time, $t_{R}$, which can be expressed as

(1) $$t_{R}=t_{0}\;\left(1+k\right)$$

where *t*_0_ is the dead time or hold‐up time of the column and *k* is the analyte retention factor, which is related to the distribution coefficient (*K*) through

(2) $$k=\frac{q_{s}}{q_{m}}=\frac{c_{s}}{c_{m}}\;\frac{V_{s}}{V_{m}}=K\frac{V_{s}}{V_{m}}$$

where $q_{m}$ and $q_{s}$ are the total mass of analyte in the mobile and the stationary phase, respectively, $c_{m}$ and $c_{s}$ are the analyte concentrations in the two phases, and $V_{m}$ and $V_{s}$ are the total volumes of each phase in the column. The retention factor is dependent on many different parameters, such as pH, temperature, and mobile‐phase composition (volume fractions of strong solvent, φ). Many equations have been described to relate *k* to one or more of these parameters and are typically referred to as retention models. Retention modeling is the process of fitting such a model to the experimental data.

LC can be performed in isocratic or gradient mode. In isocratic mode, the mobile‐phase composition is not changed over the course of the run, which means that the total mass of analyte in both phases does not change, with the result of a constant retention factor. This is not the case in gradient mode, where the mobile‐phase strength is increased over the run. This increases the total mass of analyte in the mobile phase and thus decreases the retention factor. To relate *k* to the solvent strength in gradient elution, the gradient equation has to be used. When a compound elutes before the start of the gradient, the retention time can be calculated through Equation 1, in which *k* is the retention factor at the initial organic‐modifier concentration. If a compound elutes during the gradient, the retention time can be calculated with the general equation of linear gradients [1].

(3) $$\frac{1}{B}\;\int_{\varphi_{init}}^{\varphi_{init}+B\left(t_{R}-\tau \right)}\frac{d\varphi }{k\left(\varphi \right)}=t_{0}-\frac{t_{init}+t_{D}}{k_{init}}$$

In this equation, k(φ) is the retention model (see Section 2.1), describing the relationship between the retention (*k*) and the organic modifier concentration (φ). The change in φ as a function of time (i.e. the slope of the gradient) is shown with *B* ($\varphi =\varphi_{init}+Bt$) and τ is the sum of the dwell time ($t_{D}$), the time before the start of the gradient ($t_{init}$), and the dead time (*t*_0_). In the case that the analyte elutes after the gradient, the retention time can be calculated by

(4) $$\frac{1}{B}\int_{\varphi_{init}}^{\varphi_{final}}\frac{d\varphi }{k\left(\varphi \right)}+\frac{t_{R}-\tau -t_{G}}{k_{final}}=t_{0}-\frac{t_{init}+t_{D}}{k_{init}}$$

in which $t_{G}$ represents the gradient time.

Retention modeling is mostly used to facilitate rapid and efficient method development in many modes of LC and supercritical‐fluid chromatography (SFC). The applications of retention modelling in method development can be divided in several areas. Retention models can be used to characterize newly developed stationary phases and to establish the underlying retention mechanism. In method optimization retention modelling is used to achieve better separations. In method transfer, methods developed or implemented on different systems are harmonized with the aid of retention models. Retention models are used to better understand and more‐accurately describe retention. Additionally, retention modeling is applied outside the field of chromatography, for example in pharmaceutical and environmental science, to determine the octanol–water partition coefficient ($\log k_{ow}$) of a newly synthesized product or to determine the persistence of a pollutant in the ecosystem [2, 3]. There are different strategies to perform retention modelling, depending on the aim of the study. The general form of a retention model can be described as a relation between a retention parameter and a function combining system and analyte parameters.

In a specific set of models, called linear‐free‐energy relationships (LFER), the function consists the sum of a small number (typically five) of product terms, each consisting of a system parameter ($s_{i}$) and an analyte parameter ($a_{i}$).

(5) $$\log k_{i,s}=\sum_{i=1}^{n}a_{i}\;s_{i}$$

Each term is loosely connected with a specific type of interaction between analyte and the stationary phase. Examples are the hydrophobic‐subtraction model of Snyder (HSM) [4, 5] and the model of Abraham [6, 7]. Snyder defined the stationary‐phase parameters in his model as hydrophobicity, steric hindrance, hydrogen‐bond acidity and basicity, and cation‐exchange activity [4, 5]. Abraham identified contributions of molar refraction, solute polarizability, effective hydrogen‐bond acidity and basicity, and the McGowan characteristic volume [6, 7]. In either case, the values of the system parameters depend on the values assigned to a set of reference analytes. The goal of these models is not to predict retention, but to characterize and classify stationary phases. The model does not describe the effect of the mobile‐phase composition. Characterizing analytes is laborious (requiring measurements on different columns), but characterizing systems is easy. Despite the influence of the mobile phase, system parameters are usually interpreted as column or stationary‐phase parameters and values for many stationary phases have been tabulated [8, 9].

A different angle to retention modeling is the use of quantitative structure‐retention relationships (QSRR) that are statistically derived relationships between a number of structural descriptors of an analyte and its retention [10]. Such models, based on large sets of structural parameters and retention data of many compounds, can be used to predict retention of new compounds if their structural parameters can be computed. Similar approaches have been applied to evaluate the pharmacological activity and physicochemical parameters of compounds (quantitative structure‐activity relationships, QSAR, and quantitative structure‐property relationships, QSPR, respectively). The most‐important structural descriptors are identified in the process [11].

A third approach utilizes artificial neural networks (ANNs) to describe retention for (very large) input data sets. An ANN is a computational modeling tool that is inspired by the architecture of the human brain. It consists of an input and an output layer, with one or more hidden layers in between [12], and ANN models are known to require a vast data set [13].

The final approach to retention modeling is based on (semi‐) empirical models that contain abstract parameters to describe retention. Different models have been developed for and applied to many specific modes of LC. Few input data are needed to describe retention and to predict new data through inter‐ or extrapolation. This renders the class of (semi‐) empirical models eminently useful to assist in LC method development [14, 15, 16, 17] and the remainder of this review will focus solely on such models.

Empirical retention models typically feature several parameters that are abstract (i.e. not linked to a specific interaction/mechanism in chromatography), yet which relate analyte‐ and measurement parameters (e.g. volume fraction of organic modifier, salt concentration, pH, etc.) to *k*. Often the common logarithm or the natural logarithm of the retention factor, i.e. log_10_ *k* or lnk, is used. Other variables that are not represented by the model (e.g. the stationary phase, temperature) must be kept constant at specified values for the model to remain valid. The model is typically fitted through all data points available.

With increasing computer resources, in silico techniques become much‐more attractive than exhaustive trial‐and‐error experiments for LC method development. In this review, recent developments in and applications of retention modelling will be discussed, with focus on method optimization, method transfer, stationary‐phase characterization, understanding and describing retention, and lipophilicity determination.

## 2 BACKGROUND THEORY

Several models have been developed and applied for retention modeling. In most cases, the volume fraction of modifier (φ) is the most‐important variable. Only a handful of two‐ or three‐parameter models have been used extensively, viz. the linear‐solvent‐strength model (LSS), the quadratic model (Q), the adsorption model (ADS), the mixed‐mode model (MM), and the Neue‐Kuss model (NK). Optimization programs, such as Drylab [18], PEWS^2^ [19], and MOREPEAKS [20], rely on one or more of these retention models, which are all based on the volume fraction of the modifier (φ) and are two‐ or three‐parameter models. In the following section, the models and their applications will be briefly discussed. The requirements for input data will also be discussed, such as the elution mode and the number of datapoints.

### 2.1 Models

#### 2.1.1 Linear solvent strength model

**FIGURE 1:**
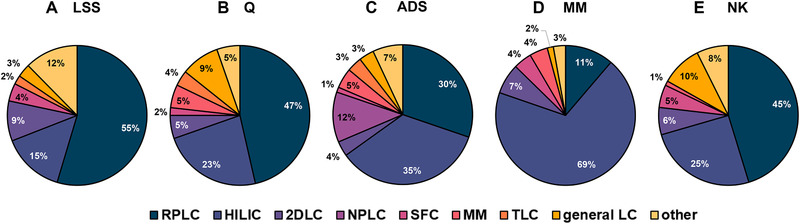
Application to different modes of chromatography for all five models. The data are based on the total number of references published from 2015 to 2020

The log‐linear model for RPLC was introduced by Snyder et al. to describe retention as a function of mobile‐phase composition (φ) [21]. It is often referred to as the LSS model (occasionally it is also referred to as the partitioning model). The common form of the model is

(6) $$\ln k=\ln k_{0}-S_{LSS}\varphi$$

where lnk is the natural logarithm of the retention factor at a specific modifier concentration, $\ln k_{0}$, often also denoted $\ln k_{w}$, refers to the isocratic retention factor of a solute in pure water, φ refers to the volume fraction of the (organic) modifier in the mobile phase, and the slope $S_{LSS}$ is related to the interaction of the solute and the (organic) modifier. The LSS model parameters can be calculated from the retention of an analyte in two isocratic runs with different φ. Of the 90 references found for the LSS model in the last six years (2015‐2020), 55% concern 1D RPLC, leaving the rest for other applications such as two‐dimensional liquid chromatography (2DLC), hydrophilic interaction chromatography (HILIC), and supercritical‐fluid chromatography (SFC), shown in Figure 1A.

#### 2.1.2 Quadratic model

Schoenmakers et al. introduced the quadratic model (Q), which can be seen as an extension of the log‐linear model with another parameter. This renders the relation between the lnk and φ convex rather than linear [22].

(7) $$\ln k=\ln k_{0}+S_{1}\varphi +S_{2}\varphi^{2}$$

In this and subsequent retention‐model equations, *S*_1_ and *S*_2_ are empirical coefficients used to describe the influence of the organic modifier on the retention of the analyte. The other parameters in the Q model are identical to those of the LSS model. Of the 45 works reported in literature that use the Q model in the past six years (2015–2020), summarized in Figure 1B, the main application is RPLC (47%). The Q model has also been investigated for describing HILIC retention (23%).

#### 2.1.3 Adsorption model

The adsorption model (ADS) was introduced by multiple researchers during the 1960s and 1970s. Soczewinksi et al. [23], Jandera et al. [24], and Snyder et al. [25] all presented the model in their work on normal‐phase LC (NPLC), and thin‐layer chromatography (TLC). The model, which was designed to account for adsorption, has also been described as the log–log model or the Snyder–Soczewinksi model [23, 24, 25, 26].

(8) $$\ln k=\ln k_{0}-n\ln\varphi$$

In this model, the *n* parameter is the so‐called solvation number, which represents the ratio of surface areas occupied by adsorbed molecules of the strong eluent component and the analyte. In contrast to the log‐linear LSS model, here lnk is linearly correlated with lnφ (log–log model). While the model was initially intended for NPLC (12%), it is now mainly used for retention modeling in HILIC (35%) and RPLC (30%) (Figure 1C).

#### 2.1.4 Mixed‐mode model

The mixed‐mode model (MM) was developed by Jin et al. to describe retention in HILIC. The idea is that it can account for both retention modes of HILIC and RPLC. The model is a combination of the LSS and ADS models [27].

(9) $$\ln k=\ln k_{0}+S_{1}\varphi +S_{2}\ln\varphi$$

Jin et al. related *S*_1_ to the solute's interaction with the solvents and $S_{2}\;$to the solute's interaction with the stationary phase [27]. The model will be discussed more extensively in Section 3.3.2. The main application of the MM model is for HILIC (69%), as shown in Figure 1D.

#### 2.1.5 Neue–Kuss model

The most recent of the five main models is the three‐parameter Neue–Kuss (NK) model that was based on another model described by Nikitas et al. [28, 29].

(10) $$\ln k=\ln k_{0}-\ln\left(1+S_{1}\varphi \right)-\frac{\varphi S_{2}}{1+S_{1}\varphi }$$

where the *S*_1_‐parameter represents the slope and the *S*_2_ ‐parameter represents the curvature of the lnk versus φ plot. When integrating this equation to obtain gradient‐elution retention times an exact solution can be found. Neue [30] suggested that this model could describe the curvature observed in lnk versus φ relationships. Later, Neue and Kuss published the following version of the model in 2010 [31].

(11) $$\ln k=\ln k_{0}+2\ln\left(1+S_{1}\varphi \right)-\frac{\varphi S_{2}}{1+S_{1}\varphi }$$

Note that the only differences between Equations 10 and 11 are the sign before and the factor 2 in the second term. In Equation 11, the *S*_1_ parameter represents the slope and the *S*_2_ parameter represents the curvature of the lnk versus φ plot. The NK model was developed for gradient RPLC, which is clear from the number of applications (45%). However, it is also often applied to HILIC (25%), as shown in Figure 1E.

### 2.2 Measurement and use of data

The way in which these empirical models can be used depends on the number and type (isocratic or gradient) of data points that are used to fit the model.

#### 2.2.1 Isocratic or gradient data

All the empirical models covered in this review have been used for optimizing both isocratic and linear‐gradient separations. Isocratic data points would need to be collected at different organic‐modifier concentrations. This can be a tedious task, since not all compounds elute at a reasonable time for every value of φ. Many organic‐modifier concentrations are often required to calculate the model parameters for every compound in a mixture. One way to get around this problem is to use gradient elution. This allows all analytes to be eluted within one run in a reasonable time. To compute the gradient‐elution retention time or the organic‐modifier concentration at the time of elution during or after the gradient, the retention model used must be numerically integrated in the gradient equation (Equations 3 and 4) [20]. Here k(φ) in Equations 3 and 4 refers to one of the above retention models. To use gradient‐elution experiments (known as scanning gradients) to establish model parameters, this process must be followed in opposite order. It is necessary to vary the effective steepness of the gradient between experiments. The effective steepness (*b*) is the product of the slope of the ln *k* versus *φ* curve (e.g., *S*_LSS_), the slope of the gradient (*B* = Δ*φ* /Δ*t*) and the hold‐up time of the column (*t*_0_)

(12) $$b_{LSS}=S_{LSS}\;B\;t_{0}=\frac{S_{LSS}BV_{0}}{F}\;$$

where *V*_0_ is the hold‐up volume and *F* is the flow rate. The effective slope can be varied by changing one of three parameters (i) the slope of the gradient (*B*), (ii) the column volume, or (iii) the flow rate.

The accuracy of prediction is influenced by the selected elution mode. An error‐analysis approach was described by Vivó‐Truyols et al. for translating gradient data to isocratic elution or vice versa [32]. The authors concluded that input data obtained using isocratic experiments yielded the most accurate predictions [32]. Isocratic elution could also be predicted using models constructed based on gradient experiments, but such models were only found accurate across limited ranges of solvent composition [32, 33, 34, 35]. Gradient retention data can be predicted from gradient‐scanning experiments [36, 37, 38], but little research has been performed on the requirements for the experimental data. In a recent paper it was shown that the prediction accuracy of the model depended on many factors, including the proximity of the slope of the predicted gradient to that of the scanning gradients, whether interpolation or extrapolation is applied, and the experimental precision [39].

#### 2.2.2 Number of input datapoints and model evaluation

When calculating the parameters of the empirical models of section 2.1, referred to as retention parameters, there is a limitation as to the minimal number of input datapoints. Generally, the number of parameters determines the number of necessary input runs. Two‐parameter models, such as ADS and LSS, need at least two datapoints for each component, while three‐parameter models, such as MM, NK, and Q, require at least three. Some of these models, for example, NK, have mostly been used with larger numbers of data [31].

Adding more parameters to a model increases the risk that the model will overfit the data. When multiple models are tested on a dataset, the number of parameters may betray the actual quality of the model. To correct for this bias, the Akaike Information Criterion (AIC) can be used, which contains a penalty when more parameters are added [40].

(13) $$AIC=2p+n\left[\ln\left(\frac{2\pi \cdot SSE}{n}\right)+1\right]$$

The AIC value is calculated upon fitting the data to the model. The sum‐of‐squares error (*SSE*) is corrected by the number of parameters (*p*) and the number of observations (*n*). A lower (more negative) value represents a better fit, thus aiding in the selection of a correct model [15, 41, 42, 43, 44, 45].

Another way of choosing between two‐ or three‐parameter models is to perform an F‐test of regression to examine whether adding a third term is significant. This test does not evaluate the goodness‐of‐fit, but only the difference between a three‐parameter model and its reduced form. From the five empirical models, the F‐test of regression can be performed on (i) the Q and the LSS models, (ii) the MM and the LSS model, and (iii) the MM and the ADS model. These three combinations have been examined by Roca et al. for modeling the retention of peptides in HILIC [37]. Baczek et al. performed an F‐test on the LSS and Q models in RPLC [46].

## 3 METHODS

**FIGURE 2:**
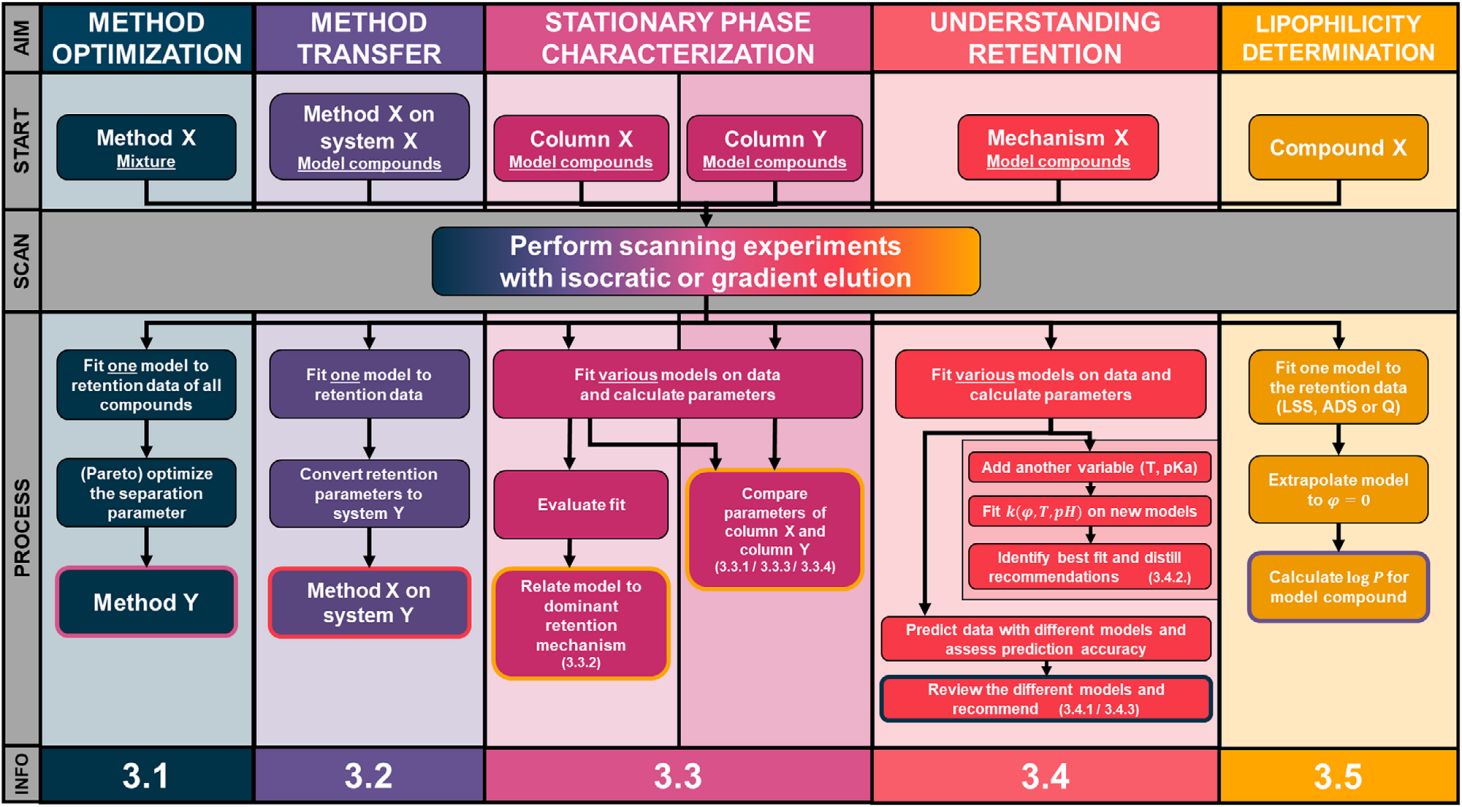
Overview of workflows of the main application domains of retention modelling in recent literature. At the top of the figure, the different aims are shown with the starting point indicated directly below. All domains will require input data which is obtained in the scan step. Next, the different processes are shown in their matching color underneath. The number of models used is indicated in the box and the final goal, whether it is a method, a number or a degree of similarity, is indicated with the colored line around the box. Beneath the workflows, the section number of the corresponding paragraph can be found. The workflows indicated in the figure are generalized and individual works may deviate from it

Upon reviewing the literature, we have identified five main domains where retention modelling is applied, and these will be discussed. These five domains and their workflows are summarized in Figure 2. For reference, the lower section of the figure contains a reference to the corresponding paragraphs in the text.

### 3.1 Method optimization

In the development and optimization of an LC method, retention modeling can be an important tool. In this section, we review recent literature on method optimization with the aid of retention modeling. The next two sections focus on 1D and 2D methods, whereas a final section addresses elaborate strategies and optimization packages.

#### 3.1.1 Optimization of one‐dimensional separations

After the mobile‐phase components and the stationary phase have been selected, retention modeling can be used to optimize separations. For example, for a separation of a vegetable oil employing a porous‐graphitic‐carbon column, Zhang et al. used the LSS model to optimize the separation with an isopropanol‐toluene gradient [47]. The authors tested the effect of the toluene fraction ($\varphi_{Tol}$) on the retention of through the *S_LSS_*‐parameter, which was found to be similar for all triacylglycerols, resulting in the same selectivity at all toluene concentrations. Conversely, differences between S_LSS_‐parameters can indicate to what extent the modifier concentration can be used to optimize selectivity. Fekete et al. reported very large *S_LSS_* values for proteins (calculated from two scanning gradients) and a tenfold decrease in retention when the organic‐modifier concentration was increased by 0.8% [48]. Such high S_LSS_ values are one of the reasons why gradient elution is indispensable in RPLC of proteins. Selectivity could be improved by serially coupling columns with increasing retentive capacity and applying multistep gradients [49, 50].

**FIGURE 3:**
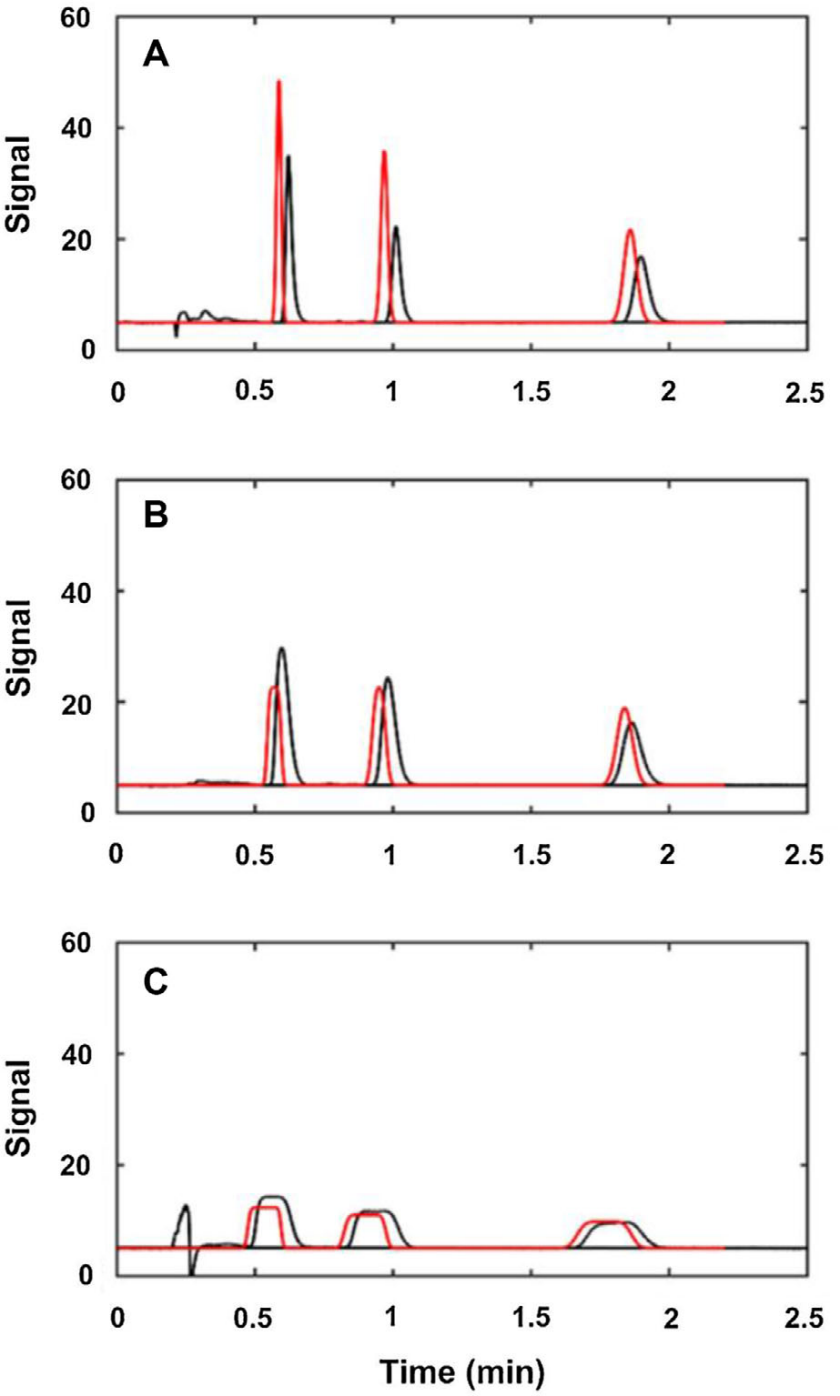
Comparison of the experimental (black) and simulated (red) chromatograms for isocratic separations of alkylbenzenes. Mobile‐phase composition was 30% water and 70% ACN. The injection volume was 100 μL of (A) 50%, (B) 70%, and (C) 90% ACN in water. Reproduced from [51] with permission

Apart from the mobile‐phase composition, the sample solvent should be considered. A large difference in elution strength between the eluent and the sample solvent may lead to incidents such as breakthrough or peak deformation [51, 52]. Jeong et al. simulated separations to study the effect of an injection with a high‐elution‐strength solvent into a weaker eluent [51]. The LSS and NK models were used for calculating local retention factors. The peak widths were predicted by considering the retention of the analyte in the injection solvent and the in mobile phase. The simulated chromatograms (red) were confirmed with experimental data (black), which is indicated in Figure 3.

Boateng et al. [53] improved a separation of three regioisomers of methoxyphenidine by optimizing the temperature, pH, and organic‐modifier concentration. Retention data were fitted using an ADS model to describe the effects of pH, the LSS or Q model for mobile‐phase composition (φ) or the van ‘t Hoff equation for temperature (*T*). By varying the three parameters at three different levels, producing a 3 × 3 input data set, an optimized separation was developed with prediction errors lower than 5%. Vaňková et al. investigated the effect of gradient steepness on peak compression using LSS retention models. They determined the mobile‐phase composition at time of elution ($\varphi_{e})$ and the corresponding retention factor ($k_{e}$) [54]. $k_{e}$ was then related to the peak width. Using this information, the peak compression by the gradient was calculated. A ratio of gradient time to column dead time of 12 was found to give the best kinetic performance for small molecules. Gritti [55] discussed peak compression more extensively and derived a new expression based on the NK model, predicting peak broadening for complex gradient programs. When the peak compression predicted by linear and non‐linear models was compared, no significant difference was observed.

Another objective of method optimization may be to maximize the sensitivity. One way to achieve this is by on‐column focusing, retaining all analytes at the column inlet by decreasing the solvent strength or by changing the temperature [56, 57]. Rerick et al. [57] used an extended form of the NK model that included the effects of temperature. The model was extrapolated to predict retention in case cooling was employed. This model will be further discussed in Section 3.4.2. Chang et al. used the retention parameters of the LSS and ADS model for their peak‐picking algorithm [58]. Clustering based on similarity of retention behavior and isotope ratios was performed. Using this strategy, the authors could rapidly identify 206 precursor ions in a complicated natural extract.

#### 3.1.2 Optimization of two‐dimensional separations

Two‐dimensional liquid chromatography (2DLC) is increasingly applied [59]. Its success can be ascribed to the combination of two different separation mechanisms (i.e. targeting different sample dimensions) and the concurrent increase in peak capacity. However, the combination of two dimensions renders method optimization a more complicated endeavor [60]. When retention modelling is applied in the development of 2D separations, the LSS model is often used to optimize the individual (gradient) dimensions [61, 62, 63, 64]. The *S_LSS_* parameter has also been used as a measure for orthogonality (i.e. the degree to which the two dimensions have different selectivities) [65, 66]. *S*_LSS_ is expected to be similar for compounds of similar molecular weight, but for a given sample it may vary. For that reason, it was decided to use the average *S*_LSS_‐parameter of all compounds in the sample of the two separations to calculate the degree of orthogonality ($O_{d}$).

(14) $$O_{d}=\gamma \cdot S_{LSS,1}\cdot S_{LSS,2}\cdot \Delta \varphi_{e,1}\cdot \Delta \varphi_{e,2}$$

It is calculated by multiplying the average *S*_LSS_ for both dimensions and the organic modifier concentration difference of the gradient of both dimensions ($\Delta \varphi_{e,1}$ and $\Delta \varphi_{e,2}$) with the correction factor with respect to the theoretical retention area (γ). This approach was developed by D'Attoma et al. [66] and applied to a RPLC×RPLC separation for peptides of Iguiniz et al. [65], which was able to separate more compounds than the prior 1DLC method. The use of LSS parameters for the degree of orthogonality is novel, although more studies are required to validate the approach.

One of the challenges in coupling two dimensions in 2DLC is the possibility of solvent mismatches, i.e. a weak solvent in one mechanism can be a strong solvent in the other. This has already been discussed to a certain extent in Section 3.1.1. Stoll et al. modelled 2D separations with gradient conditions, specifically a solvent mismatch between the injection solvent (i.e. the 1D eluent) and the 2D mobile phase [67]. The loop volumes were varied (i.e. larger loops) and different loop fillings were employed. To make these simulations, the retention behavior was assumed to be nonlinear following the NK model. Muller et al. tried to model the effect of the dilution factor, which is often applied to reduce the solvent strength of the 1D effluent, when coupling HILIC to RPLC [68]. The NK and the LSS model were used to calculate the retention factor at the time of elution ($k_{e}$) and the retention factor in the sample solvent ($k_{ss}$). The effect of several chromatographic parameters of these two retention factors are modelled to find the optimal setup for HILIC×RPLC. It was found that the predictions of $k_{ss}$ and $k_{e}$ made with LSS and NK are similar.

#### 3.1.3 Optimization programs and strategies

Many tools and software packages have been developed for LC optimization because of growing computing power. These strategies have been developed over the years with increasing knowledge of the parameters influencing retention. An example of this is the development of the Drylab, ChromSword, and PEWS^2^ method development software [18, 19, 42, 69, 70, 71]. Many of these papers are limited to RPLC, but there are three software packages that can be used for liquid chromatography in general.

Fasoula et al. developed a package of Excel VBA macros for modeling gradient retention data obtained in multilinear gradients [72]. The procedure consists of three steps: first the initial gradient retention data of each compound is fitted to a model and the parameters of that model are calculated. The model is then tested for accurate prediction of different gradients. Lastly, the optimized gradient method is determined. The package comprises ten retention models, of which the two‐parameter models, especially the ADS model, describe simulated and experimental data very well. It was found that implementing more parameters, such as the Q model, increases the accuracy of the prediction, but also consumed more computational resources. The same group published a recent paper on a more elaborate program developed in R [73]. Next to an optimized gradient separation, the software aids in other aspects of the optimization such as peak shapes and base‐line correction, and it is applied to ionized solutes too. The program contains the same 10 retention models as the Excel package of Fasoula et al. to optimize isocratic, gradient, and multigradient separations [72, 73].

**FIGURE 4:**
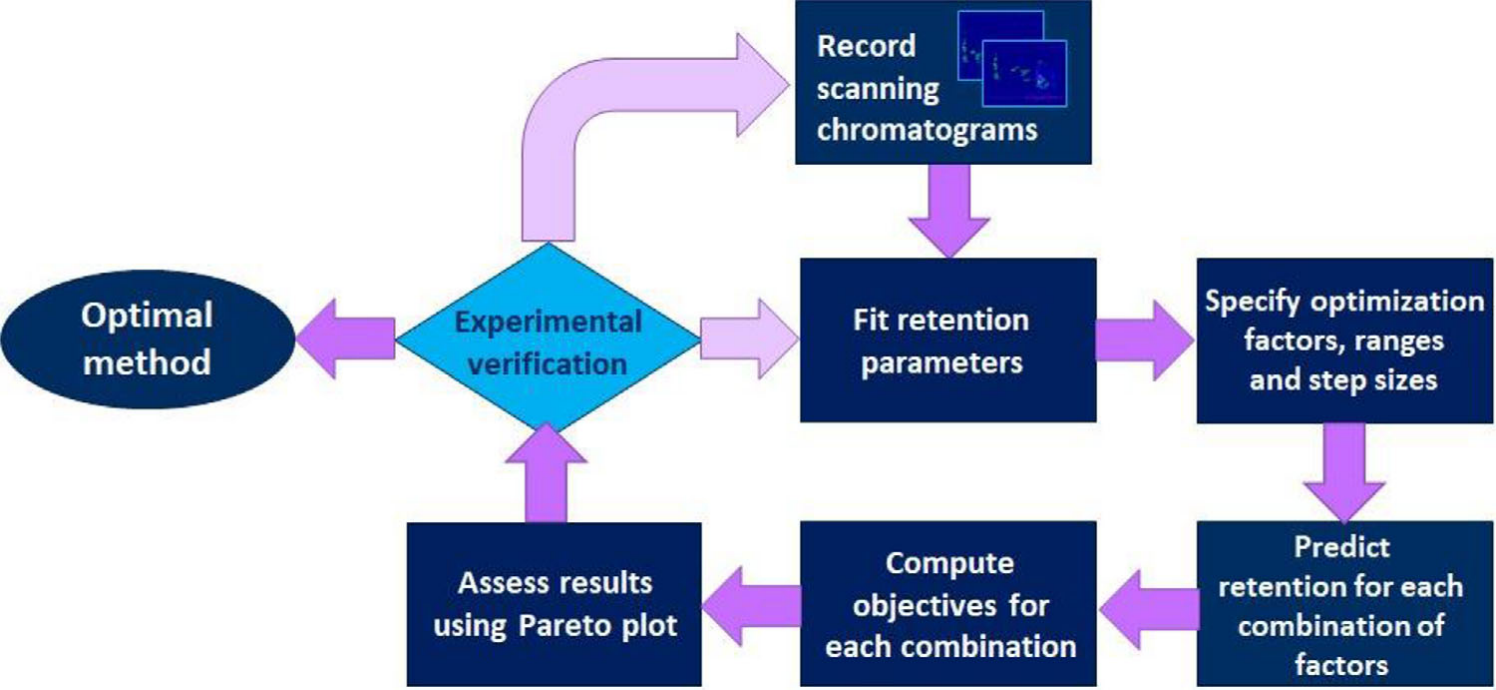
Pareto optimization strategy discussed by Pirok et al. Recorded scanning gradients are used to calculate the retention parameters by fitting the model. By specifying the ranges and step sizes in which the method can be optimized, the program predicts the retention for each component in each combination. The results are assessed in the Pareto plot and the best separation combination is verified through experimental verification. Depending on the verification, more scanning gradients are performed, or the optimal method is chosen. Adapted from [20] with permission

Pirok et al. developed an optimization program (PIOTR) for 2DLC separations in MATLAB [20]. With this program both a strong ion‐exchange separation (IEX) and an ion‐pair reversed‐phase separation were optimized. The LSS model was used for optimization in RPLC and the ADS model for optimization in IEX, replacing φ by the salt concentration [*c*]. The input data for the program is the retention data of two comprehensive liquid chromatography (LC×LC) runs, of which the gradient slopes in both dimensions differ by a factor three. The workflow of the program is shown in Figure 4. The model gave an accurate description of the retention time but could not account for the band broadening. The program also served as a good tool for peptide separation in HILIC, when the NK, the MM, and the Q model were added to the software [15, 37, 74]. Muller et al. reported on a predictive kinetic optimization tool for online HILIC×RPLC that allowed all chromatographic parameters to be optimized simultaneously within experimental restrictions [68]. The method was applied to a phenolic separation, in which the retention was modelled. Another approach was developed by Khalaf et al. for the development of RPLC separations for peptides [75]. The LSS model, the van‘t Hoff equation and an analytical solution for the mass balance on the column were combined, which was successfully applied to the separation of five different peptide mixtures. It is good to note that optimization approaches can also be conducted differently. For example, for RPLC specifically, multiple optimization algorithms have been proposed. Zhang et al. developed a new algorithm to simulate and optimize RPLC data with the aid of genetic algorithms (GA), in which the computation mimics the genetic mechanisms in all live form to adapt to the environment, and multi‐layer perceptron artificial neural networks (MLP‐ANNs) [76]. Alvarez‐Segura et al. compared the use of GAs to that of multiscale optimization (MSO), in which the level of detail in the solution is increased along the search by using subdivision schemes, for the optimization of multi‐linear gradients, simulated by the NK model [77]. The reason for this is that GAs have a hard time fine‐tuning the method. It was found that both methods yielded similar results.

### 3.2 Method transfer

Retention modeling can also be used to speed up the transfer of methods to different hardware. When gradient methods are transferred, analyst often run into the problem of different dwell volumes and gradient profiles, caused by different mixers, pumps and tubing. Bos et al. developed a response‐function‐based algorithm to determine analyte parameters with a geometry‐induced deformation correction [78]. The LSS parameters for a small set of compounds were determined on different systems with and without a correction of gradient shape, only considering the dwell time. This yielded a decrease of the inter‐system retention prediction error from 9.8 to 2.1% between the first and the second system and 12.2 to 6.5% between the first and the third system. While the study was limited to geometry‐induced deformation, the authors noted that other effects such as those induced by solvent properties, as well as solvatochromic effects still required further study.

**FIGURE 5:**
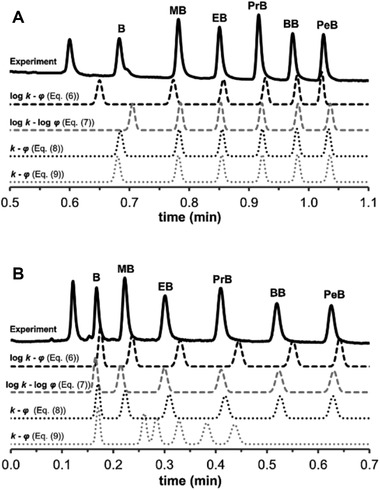
Comparison of experimental and predicted chromatogram of one‐minute gradients from (A) 0–100% ACN and (B) 50–100% ACN. The four models used are (i) LSS model, (ii) ADS model, (iii) model developed by Jandera et al., and (iv) the NK model. Reproduced from [79] with permission

Jandera et al. applied four different retention models on the prediction of the retention of a series of standard analytes in short monolithic columns with fast gradients [79]. A prediction error between 0.7 and 1.5% was found for 1 min gradients starting at 100% water for all four models, indicating the validity of the retention models to predict retention in short columns. Next to that, the ADS model provided the most accurate prediction in the fast gradients. The predicted retention of the models is compared to the experimental data in Figure 5.

**FIGURE 6:**
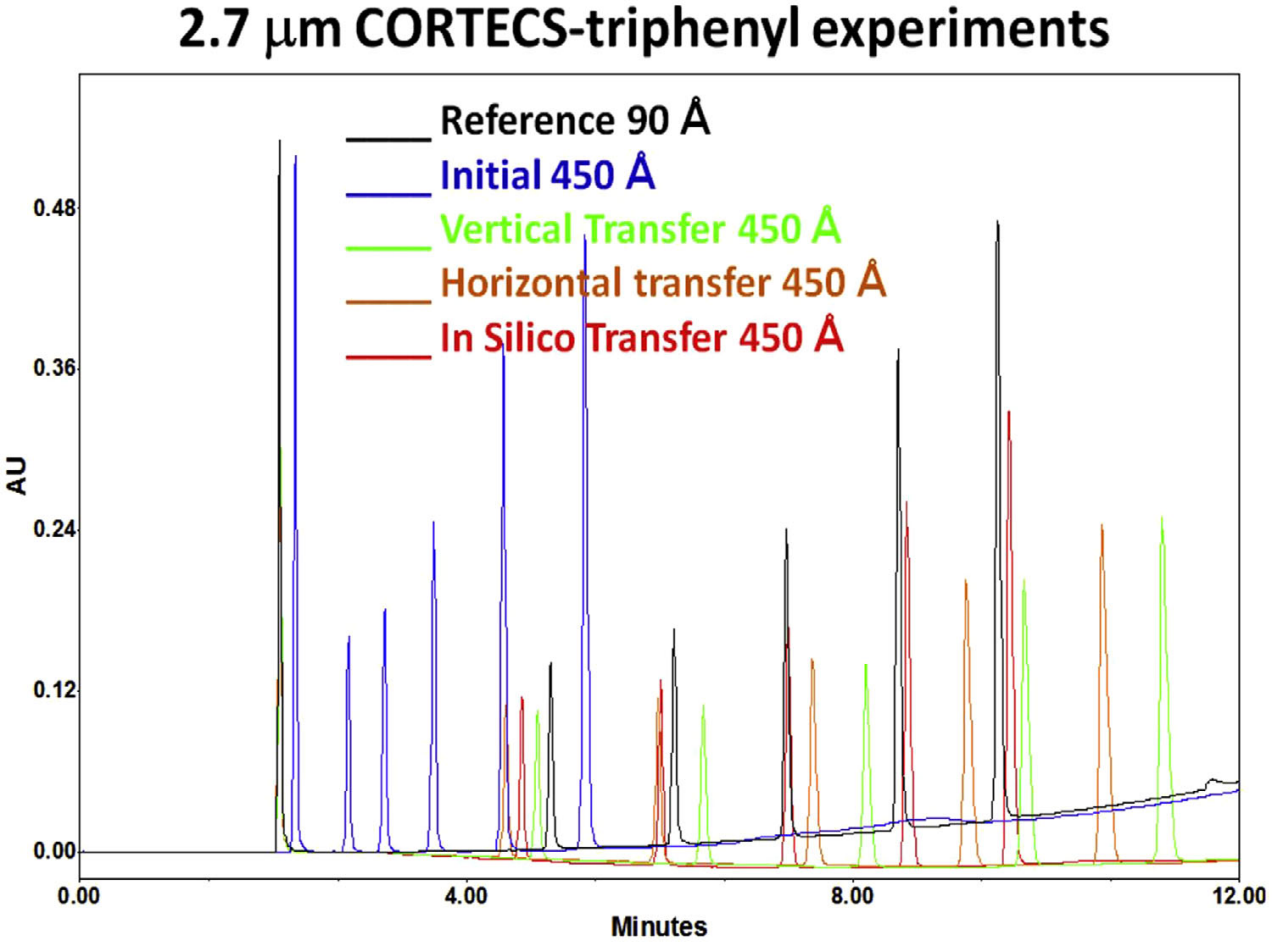
Gradient method transfer between two columns (2.7 μm CORTECS‐triphenyl) with different pore size (90–450 Å). In the figure, the reference gradient chromatogram is shown in black. The direct transfer to a column with a higher pore size is shown in blue. The three different transfer methods are indicated with green (vertical transfer), brown (horizontal transfer) and red (in silico transfer). It is visible that the black line is most similar to the red line, revealing the best gradient method transfer. Reproduced from [80] with permission

Gritti studied the transfer of a gradient method between two columns with similar particles but different average pore diameter [80]. He proposed three different gradient transfer methods to maintain the selectivity, based on either the LSS or the NK model. The first method is the “vertical” transfer, in which it was assumed that the LSS model applies for both models and that there is no change in the $S_{LSS}$‐parameter when changing columns. The retention factor on the two columns would then only vary with the change in column phase ratio ($\ln\frac{\phi_{1}}{\phi_{2}}$). The second method was referred to as the “horizontal” transfer and assumed that the new retention factor was equal to the old retention factor after shifting the eluent composition from φ to $\varphi +\Delta \varphi_{1\to 2}\;$and that the shift in eluent composition ($\Delta \varphi_{1\to 2}$) was unique for each compound. These directions refer to the shift in the lnk vs. φ plots. The third method is the in silico approach, in which the best optimal method is found by changing the gradient steepness and the starting concentration of modifier. In Figure 6, the reference chromatogram of the column with a pore diameter of 90 Å is shown with the initial chromatogram in a 450 Å pore diameter column, and the three different transfer methods. The vertical transfer was found to perform worst, while the in‐silico approach performed best. The reason for this was that the linearity of the LSS model is only an approximation.

### 3.3 Stationary phase characterization and comparison

Retention modelling is frequently used in the development and characterization of columns and stationary phases. The main part of retention modelling in column characterization is performed with models such as LFER and HSM, leading all the way to enormous column databases [81]. In these methods, the extent of different interactions occurring in a separation and the effect of these interaction on the total retention process are established [7, 82]. The models have been more extensively described in the introduction, but its exclusive application will not further be discussed in this review. Two other approaches to compare columns are classification of stationary phases by performing chromatographic tests or statistical tests and predicting retention behavior with mathematical models, requiring large amounts of data [83, 84]. Since statistical tests yield more than only retention model descriptors [85] and mathematical models often provide information on mechanical and physicochemical properties, which is often not based on any chromatographic retention data [83, 86], these two goals will not be discussed further. The fourth approach for column characterization is the application of semi‐empirical models. In this method, the fit to a model indicates which of the retention mechanisms is dominant, such as mixed‐mode [87], reversed phase [88, 89, 90], normal phase [91], but mostly HILIC [92]. In the past years, HILIC has gained popularity, leading to a better understanding of the mechanism and the influence of several parameters (see Section 3.4), which led to the development of many additional HILIC stationary phases. Stationary phases that can be used in the RPLC and the HILIC mode, depending on the level of organic modifier, have also gained in popularity.

#### 3.3.1 Column comparison

New columns or stationary phase materials are often compared to existing methods by, for example, analyzing the differences in the retention of probe compounds. A C30 bonded silica stationary phase was characterized by Vyňuchalová et al. [93, 94]. The column was compared to other RPLC columns, such as C4, C8, and C18 columns, concerning the retention of homologous non‐polar alkylbenzenes with an extended LSS model. This model included parameters for the methylene group selectivity (α) and the contribution of the end group in the series (β). In Equation 15, the constants *a* and *m* increase with the number of repeats (*n*).

(15) $$\log k=\log\beta +n\;\log\alpha =\alpha_{0}+\alpha_{1}n-\left(m_{0}+m_{1}n\right)\varphi \;$$

The retention parameters *a*_0_, *a*_1_, *m*_0_, and *m*_1_ were compared between columns, and it was found that the C30 column yielded lower parameters than the standard RPLC columns, indicating lower contributions of methylene groups and end groups and weaker effects of the organic solvent on the decrease of methylene selectivity. Similar stationary phases can be used for different purposes, for example when performing flash purification chromatography. Some manufacturers are producing flash purification stationary phases identical to the analytical stationary phase, only adapting the geometry of the column. Héron et al. compared these with a model based on the LSS model [95], where the change in S is describe with $\ln k_{w}$.

(16) $$S=p+q\ln k_{w}$$

(17) $$q=\frac{\Delta \ln\alpha_{CH2}\;}{\ln\alpha_{CH2-H2O}}\;$$

where *p* and *q* are constant for a binary solvent system. The *q*‐parameter can then be correlated to the methylene selectivity measured in pure water ($\ln\alpha_{CH2-H2O}$) and the decrease in selectivity due to an increase of the organic modifier concentration ($\Delta \ln\alpha_{CH2}\;$). As an alternative to C18 bonded phases, graphitic carbon can separate both polar and non‐polar analytes. Lunn et al. [96] compared this phase to other regular RPLC stationary phases regarding its preconcentration capability at the top of the column. The retention parameters were calculated by the NK model from isocratic retention data of small molecules on the different columns. The $\ln k_{0}$ parameter was used as a marker for its focusing ability, since the extrapolated values predicts retention in 100% water

#### 3.3.2 Classification of new fabricated stationary phases

With the increasing popularity of HILIC and mixed‐mode separations over the last years, there has been a rise in the number of stationary phases developed for these two separation modes. Most of the developments in RPLC, the workhorse of LC, are either developments of the geometrical shape of the column, such as pillar–array separations or channel shape [89, 97] (discussed in Section 3.3.4), or the addition of other separating mechanisms such as ion‐exchange, creating a mixed‐mode separation [88] (discussed in this section). Few new stationary phases for NPLC have been introduced. In 2015, Peristyy et al. investigated the retention behavior of some small molecules on synthetic polycrystalline diamond and fitted the data on the ADS model [91]. Examining the parameters calculated by the model, a higher *n*‐value (Eq. 8), the parameter that indicates the slope of the lnk versus lnφ plot, is found for the more polar compounds than for the polycyclic aromatic hydrocarbons (PAHs). This indicates hydrogen bonding on the surface of the diamond and weak dispersive forces between the PAH molecules and the flat stationary phase.

**FIGURE 7:**
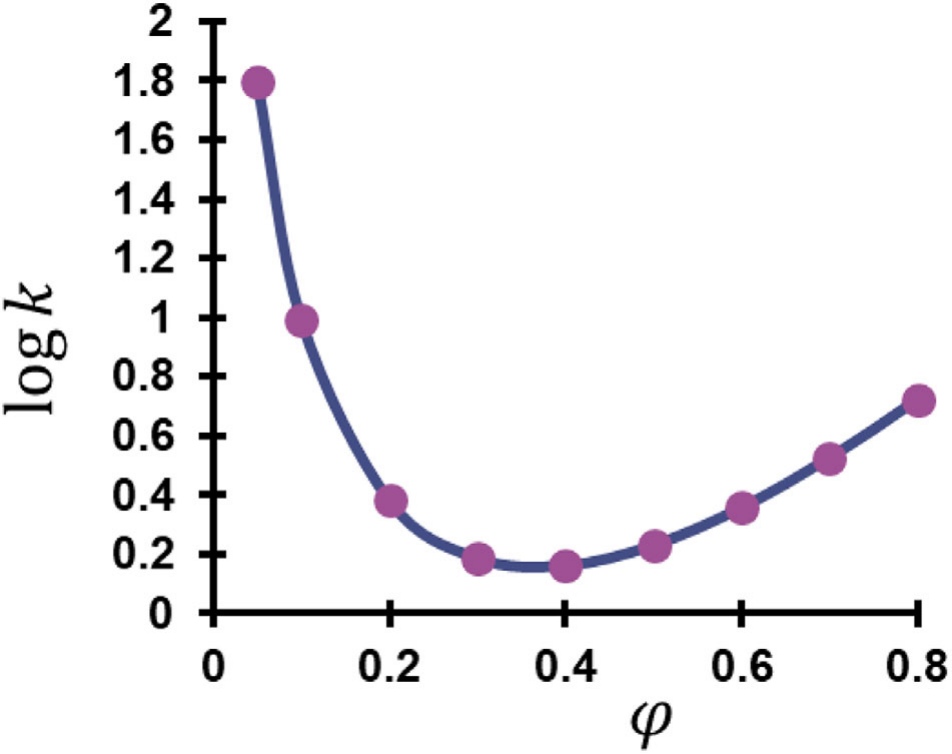
Retention behavior of gallic acid showing higher logk values at the extreme φ values. Note that φ in this figure concerns the amount of water in the mobile phase, since this is the strong solvent in HILIC. On the right side, gallic acid demonstrates HILIC behaviour and on the right side it exhibits RP behaviour. The model is shown in Equation 18 and the $\varphi_{min}$ is given by Equation 19. Plot based on the data in ref. [181]

In the past years, many different stationary phases have been developed for HILIC. To confirm the HILIC retention mechanism, the retention of some probe compounds is often measured at different levels of φ and fitted to the MM model. High regression coefficients indicate a good fit and thus confirm the HILIC mechanism, which is often indicated by a U‐profile retention (i.e. high retention at φ levels close to 0 and 1, but lower retention in between). This retention plot is shown in Figure 7.

Since the MM model is built by combining both the LSS and the ADS model, the retention data may also be fitted to these two models separately to find the dominant retention mechanism [43, 98, 99, 100]. Many of the developed stationary phases for HILIC are based on a polymeric structure, such as a hyperbranched polyethylenimine stationary phase [101] and a poly(vinyl alcohol)‐cationic cellulose copolymer [102]. Other used polymer functionalized silica stationary phases for HILIC separations are based on polyglycerol [103, 104], polyacrylamide [105], poly(vinyl alcohol) [106], and poly(glycidyl methacrylate‐divinylbenzene) [99].

With the synthesis of new stationary phases, the retention mechanism is often not limited to one specific selectivity, sometimes leading to a mixed‐mode mechanism [87]. For example, this can be based on RPLC with ion repulsion [88, 107], using the LSS model to describe the relation between retention and organic modifier. Another mixed‐mode selectivity was prepared by polymerizing a mixture of glycidyl methacrylate and 2‐dimethylaminoethylmethacrylate, yielding a dual retention mechanism of HILIC and IEX chromatography [108]. The mixed‐mode behavior was confirmed by fitting the retention data to the LSS, ADS, and MM model. It was found that acidic and neutral compounds behaved purely on an adsorption mechanism, while the basic compounds fitted best with the MM model. A perhaps more relevant and contemporary combination of selectivities is the HILIC mechanism with a RP mechanism, dependent on the level of organic modifier in the mobile phase. High levels of organic modifier induce HILIC behavior, whereas high levels of water induce RP behavior, leading to higher retention in both ends of the φ‐scale [90, 109]. The dual retention behavior can be described by the mixed‐mode model of Jin.

(18) $$\log k=a+m_{RP}\cdot \varphi_{H_{2}O}-m_{HILIC}\cdot \log\varphi_{H_{2O}}$$

Here, *a* is related to the size of the molecule and to the interaction between analyte and stationary and mobile phase, $m_{RP}$ relates to the interaction between the solutes and the solvents and $m_{HILIC}$ refers to the direct analyte‐stationary phase interaction [27, 110]. The minimum of the logk versus $\varphi_{H_{2}O}$ plots ($\varphi_{min}$), which is shown in Figure 7, can be calculated by the following equation and corresponds to the transition between RPLC and HILIC behavior of analytes. It depends on both the polarity of the sample and the stationary phase [110, 111].

(19) $$\varphi_{min}=\frac{0.434\cdot m_{HILIC}}{m_{RP}}$$

#### 3.3.3 Column comparison between SFC and LC

SFC is experiencing a renaissance in the last years and recently it was compared to LC [83]. Vera et al. published two papers in 2015 on the difference in selectivity of linear polynuclear aromatic hydrocarbons in SFC and LC [112, 113]. In the first paper the retention of the hydrocarbons was modeled with the LSS model [112]. The effect of the organic modifier on the *S*‐parameter was determined on two different stationary phases. It was found that in SFC using a similar percentage of acetonitrile reduced the retention time by half compared to the use of methanol. This led to the conclusion that retention optimization in SFC is very different from that in HPLC. In the second paper, the same selectivities in HPLC and SFC were compared and it was concluded that PAHs yield different retention between RPLC and RPSFC on the same columns [113].

#### 3.3.4 Comparing different column geometries

Besides optimizing the bonded stationary phase, there also have been improvements in the geometrical fabrication of existing stationary phases. These developments ranged from macrolevel to microlevel, which will be discussed in this order. In 2018, Gilar et al. tested the chromatographic performance of straight and serpentine microfluidic channels [97]. The LSS model was applied for the intrinsic gradient steepness ($b=S_{LSS}\times \Delta \varphi \times \frac{t_{0}}{t_{g}}$) to model the difference in gradient elution. A negative effect of turns on the efficiency was found, but this was reduced when gradient mode was employed. Gritti et al. evaluated the performance of conically shaped columns to standard cylindrical columns, where the LSS model was used for the retention in gradient elution [114]. The research indicated that, when in gradient mode, a conically shaped column could be advantageous to cylindrical columns, since it reduced peak tailing. Several scientists have devoted their attention to (the simulation of) stationary phase gradients [51, 67, 115, 116]. In one example, the isocratic retention data on the gradient stationary‐phase‐gradient columns was fitted to the NK model, which was then used to predict retention in gradient elution mode [115]. It was stated that the simulated retention fitted very well with the experimental data. This model was then applied to other stationary‐phase gradient geometries and no large differences between using a uniform mixed‐mode or a gradient column were found, but differences were found in the retention depending on the orientation of the stationary‐phase gradient (i.e. the solute retention factor either increases or decreases in the direction of the flowrate) [116].

### 3.4 Understanding, describing, and predicting retention

One of the objectives of retention modelling is to obtain a better understanding of retention mechanisms. Especially in HILIC, retention modelling can improve the understanding of the mechanism and at the same time reduce the time needed for method optimization. Models that only consider the organic‐modifier concentration, however, cannot describe the retention behavior completely. Other parameters such as buffer concentration, pH and temperature influence the separation efficiency. This section will be subdivided in three parts. The first part will consider the use of the models in section 2.1 to gain a better understanding of retention interactions. The second part focuses on new proposed models to describe more parameters in the separation besides the modifier. The third and final part will cover approaches to perform retention modeling.

#### 3.4.1 Using existing models for understanding retention

##### Reversed‐phase liquid chromatography

In many cases of retention modelling applied to RP separations the LSS model is applied. While higher‐order models often yield a better description of the data, such as the Q and the NK model, such models also require more input data and risk overfitting the data. Gilar et al. [17] investigated this problem and compared retention prediction by NK and the LSS model. The LSS parameters were calculated in three different ways: looking at the full experimental range, only on experimental data of lnk>0 and a *k* range from 1 to 30, leading to significantly different parameters. The authors concluded that nonlinear models describe the data best. The authors also recommended that if an LSS model is used it is better to omit data for lnk<0. Tyteca et al. [117] found a similar deviation from the LSS model for small molecules and peptides and found a better fit for the Q and the NK model. Next to smaller molecules, the retention data of proteins was also investigated, which led to the conclusion that, because of the very steep lnk versus φ curves, nonlinear retention behavior in proteins could not be proven. A recent study on the use of scanning gradients for RPLC optimization found that when a limited number of input experiments was desirable, good fits could be found for the two‐parameter models (LSS and ADS) [39]. However, with increasing number of sampled measurements the fit was best for the ADS model. Next to that, the research showed that the gradient‐slope factor, i.e. the ratio between slopes of the fastest and the slowest scanning gradients, which is often assumed to be at least three, is less important than the proximity of the slope of the predicted gradient to that of the scanning gradients.

For the determination of retention parameters towards the extreme values of φ, Jandera et al. [118] developed a three‐parameter model (ABM model) to allow estimation of retention in pure strong and pure weak solvent (φ = 0 and φ = 1).

(20) $$k={\left(a+b\varphi \right)}^{-m}$$

where *a*, *b*, and *m* are experimental constants depending on the solute, the stationary phase and the mobile phase [118, 119]. The authors state that in high organic‐modifier concentration, the *a*‐parameter could be neglected. This model allowed better prediction than the LSS or the ADS model and could also be used for HILIC. One of the drawbacks of the LSS model is that it cannot account for the non‐linearity, making it only applicable to the narrow linear range. Baeza‐Baeza et al. [120] extended the LSS model to include the elution strength changes with the elution degree, *g*. This parameter was assumed equal to 1 when the solvent strength followed the LSS model. It is larger than 1 when the elution strength decreased along with the organic‐modifier concentration and, reversely, smaller than 1 when it increased with the organic‐modifier concentration. In this way, it affected the linearity of the LSS model. The model could also be applied to other modes of LC.

When performing scanning experiments, it can be advantageous to reduce the time needed for the runs. Baeza‐Baeza et al. combined the accuracy of isocratic data with the speed of gradient experiments by adding solvent concentration pulses in the isocratic runs [121]. The predicted parameters were found to agree with those obtained from isocratic experiments. Gradients have also been investigated in this context, with reports focusing on the fundamental equation of gradient elution [122], non‐linear gradients [123] and pre‐elution of solute in the initial mobile phase [124].

##### Normal‐phase liquid chromatography

Because of decreased popularity, there has been little development in the field of normal‐phase liquid chromatography (NPLC) with respect to retention modeling. One study compared the slight difference in the ADS model as seen by Snyder and by Soczewinksi [125]. There exist some different perspectives about the *n*‐term in the ADS model, where Soczewinksi wrote that polar solutes and the polar solvent absorb 1:1 with the absorption sites in the silica [23], whereas Snyder defined *n* to be the ratio of molecular area of the solute required when adsorbed on the stationary phase versus the molecular area of the strong solvent [25]. Wu et al. applied these models to classic NPLC bonded phases with literature data and to the charge transfer 2,4‐dinitroanilinopropyl (DNAP) column [125]. While the Snyder model predicted better on the classed NPLC phases, the Soczewinski model predicted the charge transfer phase better.

##### Hydrophilic interaction liquid chromatography

With the potential of HILIC for the separations of highly polar and ionic compounds, the number of applications of HILIC has recently grown [126, 127]. Since the actual retention mechanism of HILIC is not yet completely understood, there have been many published reviews in recent years that attempt to describe the interactions occurring in the column [111, 128, 129, 130].

Recently, papers have focused on the optimization of a complete method, looking at different parameters such as buffers, salts, their concentrations, pH, the organic‐modifier content, temperature, and stationary phase. When optimizing the modifier content, a number of studies used the LSS model [131, 132, 133, 134, 135], the ADS model [100, 131, 132, 133, 134, 135, 136], the Q model [100, 136], and the MM model [131, 133]. Often, more than one model is used to distinguish for example between the partitioning mechanism and the adsorption mechanism [110, 111]. The effect of pH was only found to significantly influence the retention in bare silica columns, since it has a major effect on the charge of the column [100, 134]. There are several other papers that focus on the retention modelling itself. Euerby et al. [14] applied seven existing models to describe the retention as a function of the organic‐modifier concentration and three models to predict the effect of temperature. These models were added to the developed retention‐modelling program, in which the prediction accuracy could be assessed. The importance of the method parameters was ranked for retention and selectivity with statistical approaches and it was found that for retention the observed order of importance was organic‐modifier content > stationary phase > temperature≈pH≈buffer concentration and for selectivity it was stationary phase > pH > buffer concentration > temperature > organic‐modifier content. It was concluded that with gradient results, isocratic experiments could not be predicted. Cesla et al. [46] applied five existing models to the retention of oligosaccharides on different columns for which the magnitude of several mechanisms occurring in the different columns was determined. All five models fitted the retention data to a similar extent. In different studies, the LSS, ADS, Q, MM, and NK model were used by Rácz et al. [137] in Drylab and by Roca et al. [37] in MOREPEAKS. The former study concerned method development for a hallucinogenic mushroom extract on the organic modifier, the pH and the temperature for different columns. The predicted chromatograms by the Q model deviated more from the experimental results than those of the LSS model [137]. In the work of Roca et al., a tryptic digest of bovine‐serum‐albumin was analyzed and retention modelling was used to determine the best combination of column, organic modifier concentration, and additive. To confirm the selection of the model, the F‐test of regression was applied [37]. This retention modeling program was applied by Pirok et al. to separate metabolites with HILIC [15]. The ADS model was found to perform best, only requiring two scanning gradients and yielded acceptable accuracy and linearity. Next to that different stationary‐phase materials were analyzed, where the prediction accuracy in diol columns was found to be better than amide columns.

One aspect that renders the use of gradient data for the retention modeling challenging is the distortion of the gradient shape by the solvent‐delivery system [78]. Therefore, when such data is used to calculate the retention model parameters, such small errors can yield wrong φ values and thus complicate the retention prediction. Wang et al. [138] used a back‐calculation methodology for gradient imperfections and compared their HILIC results to those of RPLC. The authors concluded that column distortion plays a much more important role in HILIC retention projection compared to RPLC.

##### Mixed‐mode chromatography

When optimizing mixed‐mode separations, scientists often discuss the dual retention mechanism of RPLC and HILIC, depending on the level of organic modifier. This behavior can be described by the U‐profile retention plots, shown in Figure 7, where the minimum describes the φ where the main retention mechanism switches from reversed phase to HILIC (See Eq. 19) [129, 139]. Obradovic et al. investigated the retention of imidazoline and serotonin receptor ligands on a mixed‐mode column and were able to fit the retention data at different mobile phase concentrations to an MM model, thereby confirming the retention mechanism [140]. Balkatzopoulou et al. applied retention modelling to a mixed‐mode reversed‐phase and weak anion‐exchange column. It was found that the retention behavior could be described by a U‐profile plot, i.e., MM model, confirming the RPLC and HILIC behavior of the compounds in lower and higher organic modifier concentration respectively [141].

##### Other chromatographic modes

Retention modelling has also been applied to more uncommon forms of LC, such as micellar LC, critical chromatography (LCCC), and chiral chromatography. Since other mechanisms play a role in these types of chromatography, it is obvious that the standard retention models are not applicable to these methods. Navarro‐Huerta et al. optimized micellar LC (MLC) by isocratic or gradient elution and applied a wide range of models, some of which are developed for RPLC or specifically for MLC [142]. The most accurate predictions were found from the following model with fixed surfactant concentration:

(21) $$\frac{1}{k}=c_{0}+c_{1}\varphi +c_{2}\varphi^{2}+c_{3}\varphi^{3}+c_{4}\sqrt{\varphi }$$

where *c*_0_, *c*_1_, *c*_2_, *c*_3_, and *c*_4_ are the adjustable fit coefficients of the model. Hegade et al. applied the concept of stationary‐phase optimized selectivity (SOSLC), in which the Q model was applied to the chiral separation of enantiomers [143]. The prediction error of the retention of enantiomers on polysaccharide stationary phases was found to be within 2 and 7% for isocratic and 0 and 12% for gradient elution.

SFC often follows similar retention mechanisms as LC [83]. Vera et al. published two papers on the study of retention of polynuclear aromatic hydrocarbons on phenyl‐type stationary phases [112, 113]. These papers are discussed in Section 3.3.3. Tyteca et al. modeled SFC retention data and applied that in computer assisted method optimization [144]. The MM model, the Q model and the NK model were applied on isocratic and gradient data. The NK and MM model yielded the best retention‐prediction accuracy. The conversion of isocratic to gradient data and vice versa resulted in more difficulties due to pressure differences. De Pauw et al. [145] investigated this pressure‐related problem. Pressure and temperature definition of parameters such as fluidic CO_2_, volumes, volumetric flow rates, and mobile phase fractions, may differ between systems. The authors found that the retention in SFC could best be described through the mass fraction instead of volume fraction of the organic modifier.

#### 3.4.2 Developing new models for understanding retention

##### Reversed‐phase liquid chromatography

In many retention models, the elution mode for which the input data is measured should be the same as the preferred elution mode for prediction. For example, when recording isocratic data, the output is more reliable in isocratic mode. The same goes for gradient elution, which has even more restrictions, since the input gradient slopes should be like those predicted [39]. There have been developments to convert such data by defining the retention of analytes in gradient and isocratic elution and transferring this information to the data. This approach is referred to as the *iso‐to‐grad* approach [32]. Stankov et al. tried to apply this approach to dual‐species eluent (i.e. a combination of two organic modifiers, such as methanol and acetonitrile), and developed and tested four isocratic models with three, four, five, and eight parameters based on the Q model and the LSS model [146]. The authors deemed their prediction better than those of other models, referring to an average root‐mean square error/min of 0.743 for the compounds measured in gradient elution. Claiming a better prediction with increasing number of parameters, the authors stated that the models did not overfit the retention data. The best model was as follows:

(22) $$\begin{array}{ccc} \log k & = & a_{0}+a_{1}\cdot \varphi \left(MeOH\right)+a_{2}\cdot \varphi \left(ACN\right)\\ & & +\;a_{3}\cdot \varphi^{2}\left(MeOH\right)+a_{4}\cdot \varphi^{2}\left(ACN\right)\\ & & +\;a_{5}\cdot \varphi \left(MeOH\right)\cdot \varphi \left(ACN\right)\\ & & +\;a_{6}\cdot \varphi^{2}\left(MeOH\right)\cdot \varphi \left(ACN\right)\\ & & +\;a_{7}\cdot \varphi \left(MeOH\right)\cdot \varphi^{2}\left(ACN\right)\\ & & +\;a_{8}\cdot \varphi^{2}\left(MeOH\right)\cdot \varphi^{2}\left(ACN\right) \end{array}$$

where *a*_0_, *a*_1_, *a*_2_, *a*_3_, *a*_4_, *a*_5_, *a*_6_, *a*_7_, and *a*_8_ are regression coefficients for each analyte, solvent and column system. This model requires (at least) nine data points. Tsui et al. [147] developed a three‐parameter‐equilibrium constant stoichiometric displacement retention model for RPLC, which considers the interaction between solute‐sorbent, solute‐ACN (ACN being the mobile phase), and ACN‐sorbent, leading to the following model:

(23) $$\begin{array}{ccc} \ln k & = & -\ln\left(1+K_{SL-ACN}{C_{ACN}}^{Y}\right)-x\;\ln\left(1+K_{ACN}C_{ACN}\right)\\ & & +\ln k_{0} \end{array}$$

where $K_{SL-ACN}$ and $K_{ACN}$ are the equilibrium constants of solute‐ACN and ACN‐sorbent, respectively, $C_{ACN}$ is the free ACN concentration and *x*, *y*, and *k*_0_ are the adjustable parameters. The authors plotted both lnk versus $C_{ACN}$ and lnk versus $\ln C_{ACN}$, in which concave upward and concave downward curves were found, respectively. The developed model was able to account for the nonlinearity in the full ACN range. Unfortunately, the work was not compared to conventional retention models, and as such its performance is difficult to assess.

Gritti developed a solvent‐retention model for the description of retention in combined solvent‐ and temperature gradient LC (CST‐GLC) by combining the LSS model with the van‘t Hoff relationship [148]. In this equation, the retention as a factor of the organic modifier concentration and the temperature is written as:

(24) $$k^{\prime }\left(\varphi ,T\right)=k^{\prime }\left(0\right)e^{-S_{LSS}\left(\varphi -\varphi_{0}\right)}e^{\frac{Q_{st}}{RT_{0}^{2}}\left(T-T_{0}\right)}$$

where $k^{\prime }\left(0\right)$ is the retention factor at the initial φ and temperature, $S_{LSS}$ is the *S*‐parameter from the LSS model, $Q_{st}$ is the isosteric heat of adsorption specific for the analyte, *T* is the temperature, and *R* is the ideal gas constant. The author stated that the model described the retention of smaller compounds well over a modified range of temperature from ambient to 90°C. In the work of Wilson et al. temperature‐assisted on‐column solute focusing (TASF) was performed [56]. To model the effect of temperature on the retention, three different models were employed. Two models could be used for a fixed temperature (i.e. 1D model dependent on φ), one based on the LSS model (Equation 25) and one on the NK model (Equation 26), and a third model based on the NK model (Equation 27) included a temperature dependence (i.e. 2D model dependent on φ and *T*).

(25) $$\ln k=\ln k_{0}\;\left(T\right)-S\left(T\right)\varphi$$

(26) $$\ln k=\ln k_{0}\left(T\right)+2\ln\left(1+a\left(T\right)\varphi \right)-\frac{S\left(T\right)\varphi }{1+a\left(T\right)\varphi }$$

(27) $$\begin{array}{c} \ln k=\ln k_{0}+\frac{D}{T}+2\ln\left(1+a\varphi \right)-\left(a+\frac{D}{T}\right)\frac{S\varphi }{1+a\varphi } \end{array}$$

In these equations, *k*_0_ and $k_{0}\left(T\right)$ are the retention in pure water, *S* and S(T) describe the solvent strength, *a* and a(T) account for the curvature in the relationship between lnk and φ, and *D* indicates the effect of temperature. It was found that from the three descriptions of retention, the second equation yielded the most accurate predictions, which was calculated by measuring the retention at an organic modifier fraction of 0.05 at different temperatures. The last model (Eq. 27), which was first published by Neue and Kuss, was also used for the modelling of retention in TASF by Groskreutz et al. [31, 149]. Retention data of parabens and hydroxyphenones was fitted to the model from 12 different solvent compositions and six column temperatures, yielding an *R* ^2^‐value of 0.9996. It was claimed that the model could predict retention and shape of the peak under both isocratic and gradient elution conditions. Horner et al. evaluated three temperature‐ and mobile‐phase‐dependent retention models, of which one was Equation 27. The other two models were based on the Pappa‐Louisi partition (Equation 28) and Pappa‐Louisi adsorption (Equation 29) equations.

(28) $$\ln k=\frac{1}{T}\left(A\varphi^{2}+B\varphi +C\right)+D\varphi^{2}+E\varphi +F$$

(29) $$\ln k=A+\frac{B}{T}-\frac{\varphi \left(C+\frac{D}{T}\right)e^{E+\frac{F}{T}}}{1+\varphi \left(e^{E+\frac{F}{T}}-1\right)}$$

where both equations have six variable parameters *A*, *B*, *C*, *D*, *E*, and *F*. The model with the best fit, calculated with the AIC value (Section 2.2.2), was Equation 29. It was followed by Equation 28 and the worst fit was found with Equation 27. The authors found that Equation 27 (NK), with the lowest number of parameters, still yielded a decent estimate of the retention.

When the retention of analytes with acid‐base properties is modeled, the retention not only depends on the organic‐modifier concentration, but also on the dissociation constant (*K*). Andrés et al. developed a simplified model, based on a polarity‐parameter model [150]. This two‐parameter model, which is a simplified form of the LFER by Abraham, was originally developed for neutral compounds in RPLC [151].

(30) $$\log k=q+pP_{M}^{N}$$

**FIGURE 8:**
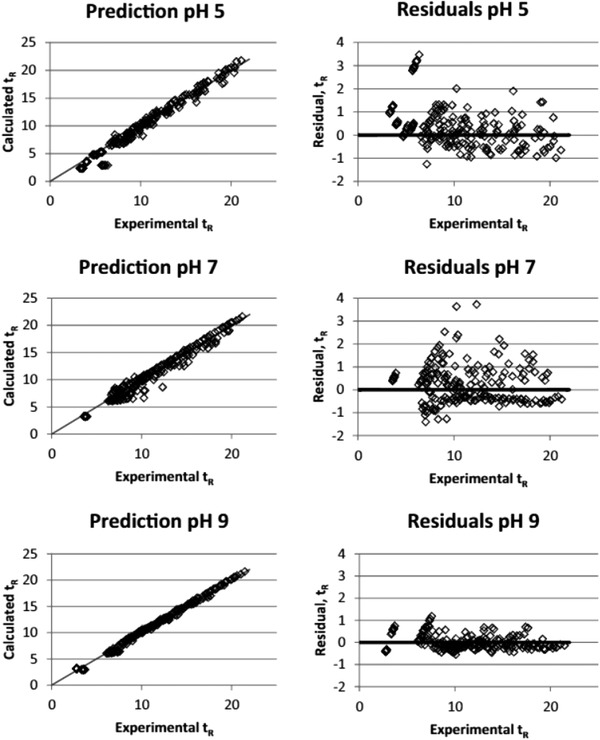
Prediction retention times, calculated with the model in Equation 31, compared to experimental retention times for pH 5, 7, and 9. On the left the two retention times are plotted for each compound and on the right the residuals of the left plot are shown. Retention time is in minutes. Reproduced from [152] with permission

In this model, $P_{m}^{N}$ describes the polarity of the mobile phase (related to the volume fraction and the different organic modifiers) and *p* and *q* are the fitting parameters. This model was extended to model retention of acid–base compounds for separations with acetonitrile and validated for separations with methanol [150, 152]:

(31) $$\log k=q+pP_{m}^{N}+\log\left[1-D\left(1-f\right)\right]$$

where $P_{m}^{N}$, *p*, and *q* are similar to Equation 30, *D* describes the ionization degree of the analytes related to the $pK_{a}$, and *f* is the ratio between the retention factors of the pure ionized and the pure neutral compound. The model was tested with different types of gradients with methanol (linear, convex, concave, and combinations of those) and at three pH values. In Figure 8 on the left, the predicted retention times for a wide range of acid‐base compounds are plotted against the experimental retention times for the three pH values. On the right, the residuals are shown. From this figure, it can be seen that a higher pH lowers the prediction error [152].

The authors claimed that the model was not size dependent, since it performed as good for small as for complex molecules [152]. Sasaki et al. found that the model of Andrés et al. gave good accuracy, but it could not model compounds that presented multiple‐curved retention behavior, i.e. molecules with multiple $pK_{a}$ values, when changing the pH [153]. In their work, pH‐modifier models were combined in an optimization program to predict the optimal separation in pH and organic modifier, based on the data of 33 runs. While the model of Andrés et al. needed information *a priori* such as the $pK_{a}$, the software package of Sasaki et al. could predict retention from the retention input data alone and needed no physiological or chemical information of the target analyte.

##### Hydrophilic interaction and mixed‐mode chromatography

As could be seen in the section “Hydrophilic interaction liquid chromatography,” HILIC is often described by the MM model. Wang et al. [154] studied the multiple interactions in HILIC further, and described the interactions between the solute, the solvent and the stationary phase into a stoichiometric displacement model for retention (SDM‐R), which is defined as follows:

(32) $$\log k=\log I-Z\log\left[W\right]$$

where logI, which represents the affinity of 1 mole amount of solute to stationary phase, and *Z* are both constant, and log[W] is the logarithm of the concentration of water in the mobile phase. The model was compared to the LSS and ADS model, and outperformed both models on the retention prediction of proteins in HILIC.

Obradovic et al. developed a novel computational approach to identify the optimal fitting models for dual retention behavior of HILIC and RPLC, typically described with a U‐profile [155]. The considered models were analyzed on their predictive ability of retention and on the accuracy of the turning point ($\varphi_{min}$). The research yielded multiple models that outperformed the standard Q and MM model, which were correlated to other parameters that are influencing the separation. These parameters were based on the average retention in HILIC mode, the average retention in RPLC mode and the average retention in the whole range of organic modifier.

#### 3.4.3 Approaches to perform retention modeling

The exact method of conducting retention modelling is not always clear. Tyteca et al. investigated fitting problems encountered when modelling retention [156]. The LSS, ADS, NK, MM, and an extended four‐parameter Neue model, as well as combinations thereof were tested on HILIC and SFC retention data. It was found that adding more scanning experiments and switching to higher‐order models could improve the fitting and modeling of the data. For highly retained compounds, the authors recommended to use very slow gradients (high $t_{G}/t_{0}$) or to start at a higher organic‐modifier concentration. Next to that, modelling retention of less retained compounds benefits from faster gradients. Another problem encountered when fitting HILIC retention data in model is that the retention in HILIC changes as a result of small changes in the mobile phase concentration. For that reason, Tumpa et al. divided the experimental space into different segments with an interpolated polynomial for each part, which is known as spline interpolation [157]. The MM, Q, ADS, and LSS model were tested in this study. The spline interpolation technique was cross validated with the standard retention modeling approach, yielding the new technique with the best values. The prediction error of the retention parameters was below 10% for all compounds.

### 3.5 Lipophilicity determination

Lipophilicity is an important parameter to describe physicochemical properties and is often used in quantitative structure‐activity relationships (QSARs) for several classes of compounds, such as environmental pollutants, pharmaceuticals, and bioactive compounds. It is generally described as the logarithm of the *n*‐octanol/water coefficient: logP. Lipophilicity is a critical parameter in drug discovery, since it plays a crucial role in determining the ADMET (adsorption, distribution, metabolism, excretion, and toxicity) of the potential candidate [158]. For successful drug discovery, drugs are assessed on their pharmacokinetic properties, such as biological half‐life and extent of protein binding, but next to that they are assessed on the delivery to these target sites. After uptake, the drugs must cross several membranes, either passively or actively. These are generally more hydrophobic and thus prefer compounds with higher lipophilicity. In recent years, the average lipophilicity value of potential drugs has increased, exposing its value and influence on the drug industry [158]. Moreover, lipophilicity plays an important role in environmental chemistry, where it is used in the estimation of bioaccumulation in plants and animals, the prediction of adsorption of pollutants in soil and sediments and the assessment of health risks of emerging contaminants [159].

There are various methods, computational and experimental, which can be divided into direct and indirect methods, to calculate lipophilicity, but there are limitations to the direct experimental methods as these (i) cost time, (ii) are labor intensive, (iii) require a lot of sample, and (iv) are often limited by the dynamic range of the detector [160]. For that reason, many indirect methods have been introduced based on separations, of which the most common approach is based on RPLC. When using RPLC as a tool, the descriptor of lipophilicity is the logarithm of the retention parameter (logk), which can be calculated from the retention time and the dead time (Eq. 1) [2]. At first, isocratic retention factors at specific organic modifier levels were used to correlate the logP to the logk, but later the authors opted that the retention factor in 100% water was more demonstrative [161]. This led to the Collander Equation, which is a linear dependence between logP and logk [162, 163]:

(33) $$\log P=a\log k_{w}+b$$

where *a* and *b* are parameters that are characteristic for the non‐polar solvent used in the chromatographic separation. Performing analyses at 100% water, which is thought to lead to major retention loss and to be catastrophic for the column life time [164], leading to large retention times of hydrophobic compounds and to very broad peaks, is omitted. For that reason, often the $\log k_{w}$ is estimated from the Snyder–Soczewinski equation (Equation 8). When calculating retention factors from 4 isocratic runs at different organic modifier levels, the $\log k_{w}$ can be extrapolated [160]. Liang et al. [165] revised the methodology of lipophilicity determination and argued to use gradients instead of isocratic runs to save time. If gradient runs are performed instead of isocratic runs, the $\log k_{w}$ found with three gradient runs agreed better with the $\log k_{w}$ determined with isocratic runs than if two gradient runs were used. Next to RPLC, Sobánska argues that TLC can serve as an alternative for lipophilicity determination, since it is inexpensive, fast, and readily available [166].

The extent to which a drug can penetrate biological membranes, such as the blood–brain barrier, cell membranes, and skin, depends heavily on the logP. A number of groups publish the lipophilicity parameters of many newly synthesized drug candidates [167, 168, 169, 170, 171, 172, 173, 174, 175, 176, 177, 178, 179, 180]. In drug discovery research, the LSS model is often used to obtain the logP, but Hawrył et al. acknowledge the concave structure of the retention plots [169]. For this reason, the Q model was used next to the LSS model to determine the intercept. The Q model gave more accurate results when acetonitrile was used as organic modifier, whereas retention data in methanol fitted better with the LSS model [1, 169]. The same quadratic relation was found by Klose et al. for wide φ ranges [177]. However, other authors found a linear relationship when using acetonitrile [174]. Sztanke et al. [167] compared modifier systems with methanol, acetonitrile, and dioxane and found that methanol systems yield the best experimental lipophilicity indices. However, the research stresses the differences and thus the complexity when performing these scanning gradients.

## 4 DISCUSSION

**FIGURE 9:**
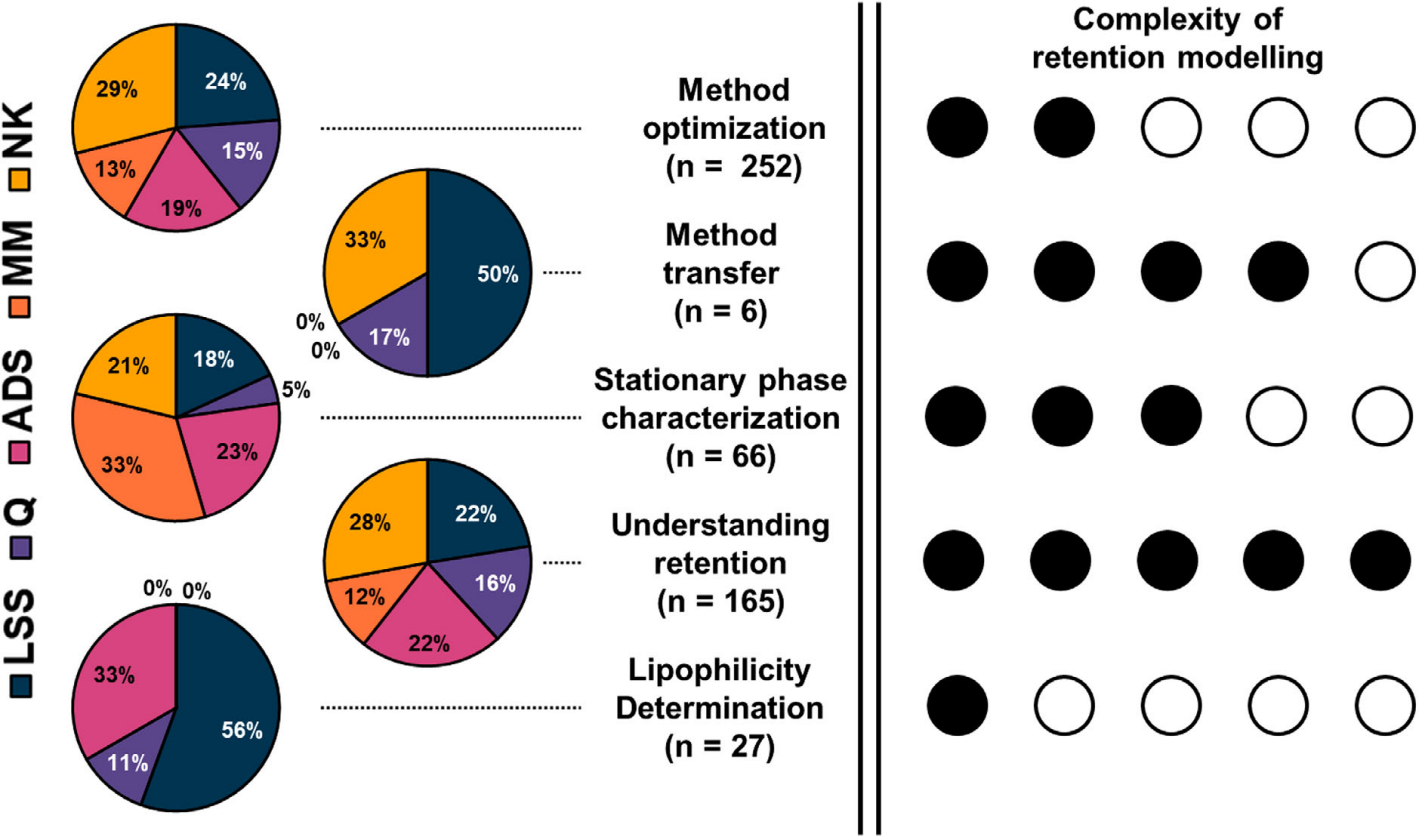
Overview of the different models applied per application. The total number of research papers is found under the title of the application. On the right the degree of complexity is given for the five different application fields

In retrospect, the application of retention modelling by means of (semi‐) empirical models has led to a better understanding of general HPLC, specifically RPLC and HILIC. It can be stated that it plays a key‐role in the different fields of application. In the published work on retention modeling, however, a clear distinction between the workflows of the different applications becomes evident, which can be seen in Figures 2 and 9. For method optimization and lipophilicity determination retention modelling is often approached as a black box. Often only one model is applied which is chosen by convention, although there seems to be enough evidence that these models have their inaccuracies. For example, with lipophilicity determination, the $\ln k_{w}$ is mostly determined by extrapolating the LSS model, while it has often been shown that there is a clear deviation from linearity, especially in the lower φ range [120]. For this reason, lipophilicity determination is ranked with the lowest complexity of the retention modelling, followed by the method optimization (Figure 9). In stationary‐phase characterization, often more models are investigated. Any good performance of a specific model is sometimes used as supporting evidence to conclude that a certain retention mechanism mainly determines the selectivity. In contrast, most work in the domain of method transfer and understanding retention use retention modeling to a much‐sophisticated extent. Researchers also appear to implement increasingly more parameters to conduct retention modeling in these latter domains, attempting to describe each contributing effect.

## 5 CONCLUDING REMARKS

The aim of this review was to provide a comprehensive overview of strategies for and applications of retention modeling. After reviewing the recent literature, we can also make a number of recommendations.

- Studies on retention modeling are typically limited to specific purposes, such as method optimization or method transfer. However, from a mathematical point of view, retention modelling is ultimately an exercise in regression statistics and insights obtained in one study should—in principle—be applicable for all purposes.
- To our surprise, numerical information on the outcome of retention modeling (e.g. residuals) are rarely reported. Also, in retention‐modeling studies, the experimental (raw) input data and relevant conditions, such as the column dead volume and the dwell volume, are often not reported. It is repeatedly unclear how specific regression results or model parameters were obtained. Unfortunately, all these factors affect the accuracy and precision of the reported retention model parameters [32, 33, 39]. Therefore, it is often not possible to reproduce or critically evaluate published work.
- The main application of retention modelling lies in the understanding, description and prediction of retention. There is currently no consensus on the quality of retention models, which frustrates the comparison and evaluation of models. Reported prediction errors range from 0.1 to 10%, but almost all authors speak of “accurate” or “good” models. Some uniformity is badly needed. Given the high efficiency of LC separations, small variations in retention times may result in large variations in resolution. Therefore, retention models for application in method development and optimization require predictions (well) within 1%.
- A potentially important application of retention modelling is method transfer. Given the enormous diversity in columns and the continuous innovation in instrumentation, method transfer is increasingly needed. Retention (model) parameters may facilitate a successful transfer of existing methods without a need for renewed method optimization.
- If better care is taken of the quality of measurements and reporting, model parameters may eventually be used as system‐independent retention data, which is an attractive proposition.

## CONFLICT OF INTEREST

The authors have declared no conflict of interest.
